# Using Genetics in Periodontal Disease to Justify Implant Failure in Down Syndrome Patients

**DOI:** 10.3390/jcm9082525

**Published:** 2020-08-05

**Authors:** Maria Baus-Domínguez, Raquel Gómez-Díaz, Jose-Ramón Corcuera-Flores, Daniel Torres-Lagares, José-Cruz Ruiz-Villandiego, Guillermo Machuca-Portillo, José-Luis Gutiérrez-Pérez, María-Angeles Serrera-Figallo

**Affiliations:** 1 Oral Surgery Department, Dentistry Faculty, University of Seville, 41009 Seville, Spain; mbaus95@gmail.com; 2 Instituto de Biomedicina de Sevilla, 41013 Seville, Spain; rgomez-ibis@us.es; 3 Dentistry in Handicapped Patients Department, Dentistry Faculty 41009, University of Seville, 41009 Seville, Spain; joserra_64@hotmail.com (J.-R.C.-F.); gmachuca@us.es (G.M.-P.); maserrera@us.es (M.-A.S.-F.); 4 Dentistry in Handicapped Patients Department, Quirón Hospital, 20012 San Sebastián, Spain; maserrera@ono.com; 5 Oral and Maxillofacial Unit, Virgen del Rocio Hospital, 41013 Seville, Spain

**Keywords:** Down syndrome, bone biology, clinical outcomes, gene expression, osseointegration, periodontal disease, dental implant, systemic disease

## Abstract

Peri-implant bone loss leading to dental implant failure does not develop in the same way across subjects who apparently present the same condition—specifically, in the case of Down syndrome patients with the same genetic disorder—given that they do not necessarily develop immune–inflammatory disorders to the same extent. Methods: This retrospective case-control study was aimed at identifying the possible genes involved in implant failure in Down syndrome patients by matching the periodontal disease variable by means of a retrospective case-control study. This process involved using the functional analysis of gene expression software Transcriptome Analysis Console (TAC, Affymetrix, Thermo Fisher Scientific, Waltham, MA, USA) and a search for the possible candidate genes involved. Focus was placed on the 92 genes related to the inflammation identified from the TaqMan™ Array Plate Human Inflammation Kit (Thermo Fisher Scientific, Waltham, MA, USA). Results: Six genes showed statistically significant results (*p* < 0.05) in our comparison. Three of them—PLCG2 (*p* = 0.0333), ALOX5 (*p* = 0.03) and LTAH4 (*p* = 0.0081)—were overexpressed in the implant reject group, and the following three were down-regulated: VCAM1 (*p* = 0.0182), PLA2G2A (*p* = 0.0034) and PLA2G10 (*p* = 0.047). Conclusion: Statistically significant differences exist in the gene expression involved in osteoclastogenesis, inflammatory response and host defensive response.

## 1. Introduction

Periodontitis cannot currently be deemed a simple bacterial infectious disease leading to the destruction of periodontal tissues. Rather, it can lead to a complex network of interactions between the host inflammatory response, the subgingival microbiota and other systemic and local modifying factors [1]. For this reason, despite the fact that the changes that occur at the level of the subgingival microbiota (dysbiosis) are necessary for periodontal disease to develop, they are not sufficient to justify the disease. This led us to think that individual susceptibility is key in the etiopathogenesis of periodontal disease.

Today, it is accepted that the accumulation of bacterial plaque represents only 20% of the risk for the development of periodontal disease [1]. In light of this, current studies need to focus on the search for the genes, proteins and metabolites involved in the inflammation-immunity mechanisms that justify the progression and/or resolution of periodontal disease [2].

We realize that scientific evidence strongly supports the association between bacterial plaque and peri-implantitis, which the European Federation of Periodontology stated in its 2019 guidelines titled “Peri-Implant Health, Peri-Implant Mucositis and Peri-Implantitis” [3]. We also know that patients with a history of periodontal disease have an increased risk of developing peri-implantitis [3]. However, we proposed the present study with the aim of identifying those genes that may be involved in the development of peri-implant bone loss leading to dental implant failure in Down Syndrome patients. We did this because we believed in the existence of an individual susceptibility factor within the same population that causes some individuals diagnosed with periodontal disease to have implant failure, whereas others have positive evolutions of the same kind.

Down syndrome is one of the most common human genetic syndromes. According to the United Nations, it has an incidence of 1/1000 and 1/1100 in newborns. Down syndrome patients present a large number of diseases that affect practically every system. At the oral level, we highlight periodontal disease, necrotizing gingivitis, chronic periodontitis and, above all, aggressive periodontitis. With respect to the latter entity, we sought to identify peculiarities with respect to the periodontopathogens and inflammation mechanisms present in patients with Down syndrome. Despite the fact that an increase has been observed in certain bacteria, such as Porphyromonas gingivalis (Pg) and Aggreggatibacter actinomycentencomitans (Aac), among others, the host response, immune–inflammatory alteration, seems to be the main cause of the development of the entity [4,5]. Alterations of immunity are well known in patients with trisomy 21.

Dental rehabilitation is necessary for those suffering from periodontal disease. In certain circumstances, the placement of dental implants is the only therapeutic option [4,5]. A patient may be selected to be an implant candidate based on factors such as the patient’s macroglossia, alveolar bone quality, poor collaboration with the treatment, tendency to develop an infection and difficulty in maintenance.

In this sense, very few case-control studies showed the results of the placement of dental implants in patients who have Down syndrome, as we argued in our earlier-published article [4]. However, Corcuera et al.’s study [6] is a notable exception given that it demonstrates how implant placement in these patients can have very adverse results. Nonetheless, the prognosis for implants in this patient category is fairly poor.

In this study, we wished to identify the genes that may be involved in the development of peri-implant bone loss by comparing two groups of Down syndrome patients with periodontal disease. One of them included patients with implant loss after two years (PD+ IR+), whereas the other group of patients presented positive implant outcomes during assessments after two years (PD+ IR-). Focusing on a gene expression study, we took as a reference the 92 genes related to inflammation that were identified using the quantification of ribonucleic acid (RNA) samples from six Down syndrome children. We also used the TaqMan™ Array Plate Human Inflammation Kit (Thermo Fisher Scientific, Waltham, MA, USA) used in the study by C.R. Silva, J.M. Biselli-Périco, B.L. Zampieri, et al. [7].

## 2. Experimental Section

This retrospective case-control study received approval from the Ethics Committee of the Virgen del Rocío University Hospital, Seville (Exp PI-0081-2016). The patients or their guardians provided their consent because the benefits obtained from the study directly impacted the patients taking part in the research. The study was descriptive and observational, as the only invasive procedure performed on each patient is the collection of a small amount of blood and a dental examination.

### 2.1. Sample and Groups

The two study groups were made up of Down syndrome patients diagnosed with periodontal disease (PD); the first group (1) also presented implant rejection (periodontal disease plus implant rejection, or PD+ IR+), whereas the second group (2) showed positive assessments of their implants (PD+ IR-) after two years. The criterion used for determining that a patient was suffering from periodontal disease is the one that Bassani et al. [8] developed. In accordance with this criterion, periodontal disease was defined by the presence of three or more teeth with one or more sites with clinical attachment loss greater or equal to 3 mm, plus the existence of BOP (Bleeding on Probing) in said sites. It is worth highlighting that during rehabilitation with implants, peri was inactive (BOP negative), or patients had previously lost all of their teeth. Within the concept of implant rejection, we included implant failures resulting from a lack of osseointegration, as well as implants that had to be removed for presenting bone loss greater than 50% of its length in the follow-up period, whether or not the implants were accompanied by perimplantar infection.

The exclusion criteria used include the following:

- Patients without Down syndrome
- Patients receiving treatment that could have repercussions for bone metabolism
- Patients with untreated periodontal disease

Four patients were included who had periodontal disease and implant rejection compared with six patients who had periodontal disease and positive implant outcomes, thus leading to a total of 10 patients in the study. Demographic and clinical variables were taken from medical records, with all essential details used in the verification process.

### 2.2. Sampling and Total RNA Isolation

At the time of the examination of the patients who were selected and included in this study, a sample was taken comprising two blood draws from the median cubital vein per person in PAXgene™ (5 mL) tubes (Qiagen N.V., Hilden Germany), reference 762165 (100 tubes), with the final aim of extracting RNA. The transfer of the samples to the processing center was undertaken in refrigerated conditions (2–8 °C) and never took more than three days.

The extraction of the RNA sample was undertaken using a QIAgen PAXgene Blood miRNA Kit (Qiagen N.V., Hilden Germany), reference 763134, at an automated QIAcube (Qiagen N.V., Hilden Germany). Subsequently, a database was created for the samples detailing, inter alia, the RNA quantification data.

First, RNA concentrations were quantified using visible light spectroscopy with NanoDrop 2000c equipment (Thermo Fisher Scientific, Waltham, MA, USA) to ensure the correct processing of these before storage. Second, a much more accurate measurement was taken using fluorescence and Qubit 3.0 equipment (Thermo Fisher Scientific, Waltham, MA, USA) to study gene expression, the results of which were added to the database.

### 2.3. Functional Analysis of Expressed Genes

The experimental approach was one of the last steps taken in the study of gene expression to find candidate genes in metabolic pathways that would be of interest. The GeneChip^®^ Scanner 3000 platform (Thermo Fisher Scientific, Waltham, MA, USA) was selected, and the chips chosen were Clariom S solutions for a human, mouse and rat, with more than 20,0000 genes annotated for measuring expression levels (Thermo Fisher Scientific, Waltham, MA, USA).

The RNA selected was amplified and hybridized using the GeneChip^®^ WT PLUS Reagent Kit (Thermo Fisher Scientific, Waltham, MA, USA). Amplification was performed from an initial total of 55 g and was undertaken in accordance with the manuals in the GeneChip^®^ WT PLUS Reagent Kit (Thermo Fisher Scientific, Waltham, MA, USA).

Finally, analysis was undertaken by normalizing and using the robust multi-array method.

### 2.4. Statistical Analysis from the 96-Plex Card Genes

An analysis of the various gene expressions was undertaken using Transcriptome Analysis Console (TAC, Affymetrix, Thermo Fisher Scientific, Waltham, MA, USA) software. Genes that were used as a reference for the search from the results obtained from our study are the 92 genes related to the inflammation of the 96-plex gene card (Table 1) that were quantified from the RNA samples of six Down syndrome children using the TaqMan^®^ Array Plate Human Inflammation Kit (Thermo Fisher Scientific, Waltham, MA, USA) [7]. Values less than 0.05 were deemed statistically significant and are the ones shown in this study.

## 3. Results

### 3.1. Gene Expression Analyses

Of the 92 genes used as a reference from the above-mentioned study [7], only six showed statistically significant differential gene expression (*p* < 0.05) when our two study groups (PD+IR+ vs PD+IR-) were compared using Transcriptome Analysis Console (TAC, Affymetrix, Thermo Fisher Scientific, Waltham, MA, USA) software. Three of the genes—PLCG2, ALOX5 and LTAH4—showed up-regulated results, whereas the VCAM1, PLA2G2A and PLA2G10 genes appeared to be down-regulated (Table 2).

None of the genes of PLCG2, ALOX5, LTAH4, VCAM1, PLA2G2A and PLA2G10 showed statistically significant differential expressions when the study groups were modified into patients with periodontal disease and implant rejection versus patients without periodontal disease and with positive evolutions of their implants (PD+IR+ vs. PD-IR-). The study group in our previously published article [5], and, similarly, genes that were expressed differentially in PD+IR+ versus PD-IR-, ITGB2, TNFSF13B, ANXA3 and ANXA5 did not show any alterations in the new comparison of this study (PD+IR+ vs. PD+IR-). This leads us to think that, when it comes to matching the periodontal disease suffering variable, the genes most closely related to its development were expressed in similar fashions in both study groups, thus highlighting the genes involved in other metabolic pathways. This might explain why one group experiences implant failure but the other does not, even though they both suffer from periodontal disease.

### 3.2. Functional Analyses of Differentially Expressed Genes

The six genes that showed statistically significant differential expressions were analyzed using the Online Mendelian Inheritance in Man^®^ (OMIM) database (Johns Hopkins University, Baltimore, MD, USA), an online catalog on human genes and genetic disorders, and the information was completed using data available in the Kyoto Encyclopaedia of Genes and Genomes (KEGG), as well as the literature available in PubMed (Table 3).

Of the six genes, only PLCG2 directly takes part in the osteoclastic differentiation pathway. However, it does not appear to be the only gene that codifies a molecule involved in osteoclastogenesis, as ALOX5 and LTA4H seem to be involved in inducing the formation of osteoclasts mediated by RANKL and other pathways, such as MAPK and NF-kB, which is argued later.

On the other hand, VCAM1, PLA2G2A and PLA2G10, down-regulated genes, maintain, amongst other functions, close relationships with the defensive host response in the face of bacterial infection, inflammation and immune response regulation/suppression situations.

## 4. Discussion

In this study, we attempted to discover the underlying reason for the high rate of implant failure that we found in Down syndrome patients. This is important given that, on occasion, the only therapeutic option we have for rehabilitating occlusion and therefore improving their quality of life is the placement of dental implants.

In the article that our study group published earlier [5], the conclusion reached was that despite that both groups suffered from the syndrome, patients who were diagnosed with periodontitis presented certain degrees of individual susceptibility stemming from the differential expressions of genes such as TNFSF13B, ITGB2, ANXA5 and ANXA3, as well as their involvement in immune and/or inflammatory disorders. This supports the fact that even though they are individuals with the same genetic disorder, they do not necessarily develop inflammatory disorders, such as periodontitis, to the same degree.

Now let us take one step further: just as periodontal disease develops in different degrees of severity in individuals who apparently share the same condition, why do some patients who present, by definition, disorders in the immune–inflammatory system, such as Down syndrome, present implant failure whereas others maintain functionality?

Considering the above, in this study, the comparison of groups was decided in which all individuals that presented active periodontal disease or clear signs of disease affecting peri-implant health in the case of edentulous patients, two years after the diagnosis of the implant failure or positive implant outcome. Of the 92 genes related to inflammation that are presented in the 96-plex card gene [7], only six genes provided statistically significant (*p* < 0.05) results.

Phospholipase C gamma 2 (PLCG2) is the only gene in our results that showed direct involvement in osteoclast differentiation. PLCG2 was overexpressed in the implant rejection group with *p* < 0.033. PLCG2, the protein codifying the gene, regulates the route of osteoclastogenesis through its interaction with proteins ITAM and GAB2. Its elimination results in an in vivo osteopetrotic phenotype in mice through the incapacity to form mature osteoclasts, without any difference with respect to osteoblasts and the rate of bone formation or mineral apposition [9] observed. This then leads us to understand that it is an intrinsic defect in the osteoclast lineage. We realize that PLCG2 elimination can result in an osteopetrotic phenotype [9,10] with an increase of more than three times the percentage of trabecular bone volume, a smaller number of osteoclasts per bone perimeter, and a larger number of bone trabeculae, as well as those that are larger in thickness with less space between them. Provided that we are talking about an increase in PLCG2, as is the case in our results, we could speculate about an opposite situation and therefore a possible explanation for peri-implant bone loss by the osteoporotic phenotype. Furthermore, this is supported by the fact that a mutation in PLCG2 provokes an augmented and sustained response from calcium [11], which perpetuates the activation of NFATc1 [10,12,13], a key transcription factor for the expression of specific osteoclast genes. In our results, greater osteoclastogenesis could be justified by an increase in NFATc1 activation as a result of a greater flow of calcium sustained by a greater expression of PLCG2.

In addition, PLCG2 takes part in the NF-kB signalling pathway, specifically in the B cell receptor signalling pathway (BCR). Changes in this pathway result in stronger BCR signals due to increased calcium responses, with a direct impact on the development of B cells, which can lead to a breakdown in tolerance [11]. Therefore, we might be led to believe that overexpression in our results for PLCG2 would give rise to an osteoporotic phenotype through direct participation in osteoclastogenesis. However, we would also be talking about an impaired inflammatory situation characterized by severe inflammation and autoimmunity, through an increase in B cell activity. This, in turn, would amplify osteoclast recruitment at the inflammation site.

Likewise, macrophage inflammatory proteins (MIP) take part in osteoclast recruitment and development at bone resorption sites. Specifically, MIP-1D increases the phosphorylation of PLCG2 and therefore its activity [12]. Consequently, again, we highlight this enhanced osteoclastogenesis response not only through the inherent overexpression of PLCG2 but also through the direct action of other cytosines, such as MIP-1D.

ALOX5, Lipoxygenase-5 and LTA4H, LTA4 Hydrolase, are jointly analyzed because they form part of the same pathways. The main function of ALOX5 is to transform fatty acids, such as arachidonic acid (AA), into leukotrienes, specifically into Leukotriene A4. LTA4H, in short, transforms LTA4 into LTB4, Leukotriene B4. It is well known that leukotrienes play an important role in inflammation, and one of the actions attributed to LTB4 is inhibition in bone formation and stimulation in bone resorption [14]. In our results, not only is ALOX5 overexpressed but also LTA4H is overexpressed. Therefore, we are talking about a joint increase in LTA4 and LTB4.

In a study [14], it was observed that when it came to inhibiting ALOX5, RANKL-induced osteoclast formation was inhibited without causing cell toxicity. The prevention of osteoclast formation and LPS-induced bone resorption lacunae [14,15] is shown in vivo. One can observe an improvement through the restoration of mineral bone density; an increase in trabecular bone volume; and a greater trabecular thickness, number and separation. In our results, not only was ALOX5 overexpressed, but we also encountered a situation in which LPS was present given that periodontitis predominantly presents microbiota of Gram-negative bacteria. This is an unfavorable situation because it has a gene that seems to boost osteoclast activity. In addition, bacterial action drives the latter.

As mentioned earlier, the expression of NFATc1 is decisive for osteoclastogenesis. Its regulation may occur via the calcium signalling pathway, where, in our results, PLCG2 was overexpressed. Its regulation may also occur via the MAPK and NF-kB pathways, where the literature supports the influence of ALOX5 on NFATc1 expression [15]. Consequently, if our results showed that ALOX5 was also overexpressed, we could say that there was a greater expression and therefore activation of NFATc1 through all of the possible osteoclastogenesis pathways.

Similarly, LTB4, which is considered to be the most pro-inflammatory metabolite of ALOX5 [16], is obtained from LTA4 through the action of LTA4 hydrolase (LTA4H), a gene that was also overexpressed in our results. LTA4H takes part in chemotaxis, the production of reactive oxygen species, and in the production of IL1, IL6 and IFNG. LTB4 is augmented in crevicular fluid and in the saliva of patients with periodontal disease [16], and it is associated with a greater level of severity of the disease [17]. Likewise, it has been related to improvement in the differentiation and activity of osteoclasts that negatively affect bone turnover without the need to increase the number of osteoclasts. It has also been related to reducing the differentiation and activity of osteoblasts, as well as improving the calcium flow [16,17,18,19]. This is supported by ALOX5 inhibition [16,17], where the inhibition of bone resorption has been observed via a significant reduction in the number of osteoclasts, as well as a decrease in the severity of the inflammation, due to a significant reduction in the number of polymorphonuclear (PMN) and mononuclear cells. This is associated with a discreet increase in IL-10, an anti-inflammatory cytokine, probably due to a lower level of the production of LTB4 [16], even in the presence of an infection with *Aggregatibacter actinomycetemcomitans* [17]. In our results, LTA4H was overexpressed, which could be understood as an increase in LTB4 in PD+IR+ patients compared with PD+IR-. This is associated with an increase in resorption at the bone level, as well as a prolonged and marked inflammatory response at the immune level. With regard to this latter point, it should be emphasized that in patients with implant rejection, it has been observed that the gene codifying IL-10, an anti-inflammatory cytokine, is down-regulated.

It appears that all of our findings point to the study patients who presented implant failure exhibiting prolonged and marked osteoclastogenesis compared with the group presenting positive implant outcomes. With both groups featuring Down syndrome patients, the groups were faced with the same underlying genetic condition and the same oral pathological condition. Both groups suffered from active periodontal disease and therefore chronic inflammation.

Furthermore, VCAM1 is the gene that codifies vascular cell adhesion protein 1, and it mediates lymphocyte, monocyte, eosinophil and basophil adhesion to the vascular endothelium. Besides taking part in the signal transduction of leukocyte endothelial cells, it appears to show activity on osteoclasts, this time through skeletal stem cells. These cells have been defined as specific skeletal stem cells, deemed necessary for bone development and remodeling. They present potential for inducing osteoprotegerin (OPG) expression in inflammatory environments by suppressing osteoclast formation and therefore bone resorption. They act in synergy with ICAM-1 and VCAM-1, which are necessary for capturing osteoclast precursors. This enables OPG to work in proximity to the cells to inhibit RANKL, inter alia [20]. In our results, we observed a lower level of expression of VCAM-1 in the implant rejection group. We could observe a reduced efficiency of OPG for inhibiting osteoclastogenesis in an inflammatory environment, as is the case with periodontal disease.

However, osteoclastogenesis is not the only metabolic pathway that seems to be impaired and related to implant failure in Down syndrome patients. Focusing more on the immune–inflammation response, VCAM-1 is deemed important in mesenchymal stem cell (MSC)-mediated immunosuppression. This is because for MSCs to exert their powerful immunosuppressant activity through NO production in the mouse immune system, lymphocytes must be close to the MSCs. Curiously, in the human immune system, MSCs used indoleamine 2,3-dioxygenase (IDO) instead of NO to suppress the immune response and the cell–cell contact mediated by the adhesion molecules necessary for immunosuppression [21]. If we observed down-regulated VCAM-1 in our results, one might consider an inflammatory response perpetuated by lower T-cell-mediated immunosuppression at the inflammation site.

Finally, we focus on PLA2G2A and PLA2G10 genes, which belong to groups II and X of phospholipase A2, respectively. PLA2G2A codifies type IIa secreted phospholipase A2 (sPLA2-IIA), which is considered to be a “natural host antibiotic,” as it is an antimicrobial polypeptide [22]. sPLA2-IIa may act independently against Gram-positive bacteria and is considered to be the most powerful tool for eliminating this type of bacteria, followed by group X-secreted phospholipase A2 (sPLA-X). sPLA2-IIA can be used in combination with other defense systems, bactericidal/permeability-increasing protein (BPI), a complement system, lysozymes, etc. [23,24]. sPLA2-IIA’s concentration increases in the serum of patients with sepsis, which points to a fundamental role in the immune response [24]. Note that sPLA2-IIA increases its expression after inflammatory stimuli or bacterial invasion. Curiously, sPLA2-IIA expression increases up to 3900 times when it is exposed to *Porphyromonas Gingivalis* (Pg) [24,25], which suggests that Pg acts by directing change in the host microbiota, instead of inducing inflammation directly. This unleashes a chronic inflammatory response that leads to a greater destruction of periodontal tissue.

Continuing with the bactericidal properties of sPLA2-IIa and sPLA-X, in humans, it is observed that group IIa has the greater level of bactericide action, as it is very efficient (it kills ≅ 99% of bacteria) in minimum concentrations (0.5 μg/mL), followed by group X, which kills ≅ 99% of bacteria but at a concentration of 50 μg/mL [26]. If PLA2G2A and PLA2G10 were down-regulated in our results, greater susceptibility to infections by impaired natural antimicrobial action could be contemplated. Furthermore, this leads us to think about what PLA2G2A expression and PLA2G10 expression would be like if we were to compare Down syndrome patients with a non–Down syndrome control group. Would it be one of the possible causes of susceptibility to infection in patients who suffer from the syndrome?

We recognize the need for a subsequent study on gene validation to determine the accuracy of the genetic alterations found, given the sample size.

## 5. Conclusions

Our study showed how patients with Down syndrome who were diagnosed with active periodontal disease presented with different evolutions compared with those rehabilitated using dental implants. Despite the fact that all of the patients in the study suffered from the same genetic disorder and the same immuno-inflammatory disorder by definition, the study group that showed positive evolutions of the implants (PD + IR-) seemed to be able to stabilize the lesion, whereas the group with implant failure (PD + IR +) exhibited differential expressions of genes that participated in the different activation routes and progression of osteoclastogenesis. This seems to lead to an accelerated loss of peri-implant bone support directed by the deterioration of the immune–inflammatory response. Therefore, we could call it a genetically susceptible group that causes the condition to progress and leads to the clinical manifestation of peri-implantitis. This leads us to accept that the inflammatory response of the host drives the pathological process, to a great extent, not only for the periodontal disease but also for the peri-implantitis. Therefore, it also leads to the understanding that the inflammation of the periodontal and peri-implant tissues leads to the destruction of local tissue.

Recognizing the importance of being able to modulate the inflammatory response and determining the patient’s genetic circumstances may represent a new treatment opportunity.

Finally, we note that the differential gene expression studied in this article does not occur in chromosome 21, which highlights the genetic complexity that underlies Down syndrome.

## Figures and Tables

**Table 1:** A total of 92 genes related to inflammation, and four reference genes (ª) from the 96-plex gene card [7].

18Sª	GAPDHª	HPRT1ª	GUSBª	A2M	ADRB1	ADRB2	ALOX12	ALOX5	ANXA1	ANXA3	ANXA5
**KLK3**	BDKRB1	BDKRB2	CACNA1C	CACNA1D	CACNA2D1	CACNB2	CACNB4	CASP1	CD40	CD40LG	CES1
**LTB4R**	MAPK14	NR3C1	HPGD	HRH1	HRH2	HTR3A	ICAM1	IL1R1	AL2RA	IL2RB	IL2RG
**IL13**	ITGAL	ITGAM	ITGB1	KTGB2	KLK1	KLK2	KLKB1	KNG1	LTA4H	LTC4S	MC2R
**NFKB1**	NOS2	PDE4A	PDE4B	PDE4C	PDE4D	PLA2G1B	PLA2G2A	PLA2G5	PLCB2	PLCB3	PLCB4
**PLCD1**	PLCG1	PLCG2	MAPK1	MAPK3	MAPK8	PTAFR	PTGDR	PTGER2	PTGER3	PTGFR	PTGIR
**PTGIS**	PTGS1	PTGS2	TBXA2R	TBXAS1	TNF	TNFRSF1A	TNFRSF1B	VCAM1	IL1R2	PLA2G7	PLA2G10
**PLA2G4C**	IL1RL1	HTR3B	TNFSF13B	CYSLTR1	HRH3	PLA2G2D	IL1RAPL2	KLK14	PLCE1	KLK15	LTB4R

**Table 2:** Results of the differentially expressed genes across the two study groups: Down Syndrome Patients with Periodontal Disease and Implant Rejection (PD+IR+), and Down Syndrome Patients with Periodontal Disease and Positive Outcome of Implants (PD+IR-). (AVG = Average.).

GENE	GENE ID - OMIM	GENE NAME	PD+IR+ AVG (Log2)	PD+IR- AVG (Log2)	PD+IR+ Standard Deviation	PD+IR- Standard Deviation	Fold Change	*p*-Value	Chromosome
**PLCG2**	* 600220	Phospholipase c, gamma-2	12.32	11.85	0.41	0.39	1.39	0.0333	chr16
**ALOX5**	* 152390	Arachidonate 5-lipoxygenase	11.24	10.29	0.6	0.9	1.94	0.03	chr10
**LTA4H**	* 151570	Leukotriene a4 hydrolase	14.11	12.76	0.81	0.67	2.55	0.0081	chr12
**VCAM1**	* 192225	Vascular cell adhesion molecule 1	5.51	6.27	0.22	0.48	−1.69	0.0182	chr1
**PLA2G2A**	* 172411	Phospholipase A2, group IIa	3.95	4.74	0.27	0.49	−1.73	0.0034	chr1
**PLA2G10**	* 603603	Phospholipase A2, group X	5.51	6.02	0.21	0.46	−1.42	0.047	chr16

**Table 3:** Metabolic pathways in which each of the genes takes part with statistically significant results in our study. In the pathways column, only the pathways that are of interest in this study have been shown. The rest of the pathways in which the genes take part may be found at https://www.kegg.jp.

GENE	PATHWAYS (KEGG)		*p*-VALUE	FOLD CHANGE
PLCG2	ko04020	Calcium signalling pathway	0.0333	1.39
	ko04064	NF-kappa B signalling pathway		
	ko04380	Osteoclast differentiation		
ALOX5	ko00590	Arachidonic acid metabolism	0.03	1.94
	ko01100	Metabolic pathways		
LTA4H	ko00590	Arachidonic acid metabolism	0.0081	2.55
	ko01100	Metabolic pathways		
VCAM1	ko04064	NF-kappa B signalling pathway	0.0182	−1.69
	ko04514	Cell adhesion molecules (CAMs)		
	ko04668	TNF signalling pathway		
	ko04670	Leukocyte transendothelial migration		
PLA2G2A	hsa00564	Glycerophospholipid metabolism	0.0034	−1.73
	hsa00565	Ether lipid metabolism		
	hsa00590	Arachidonic acid metabolism		
	hsa00591	Linoleic acid metabolism		
	hsa00592	alpha-Linolenic acid metabolism		
	hsa01100	Metabolic pathways		
PLA2G10	hsa00564	Glycerophospholipid metabolism	0.047	−1.42
	hsa00565	Ether lipid metabolism		
	hsa00590	Arachidonic acid metabolism		
	hsa00591	Linoleic acid metabolism		
	hsa00592	alpha-Linolenic acid metabolism		
	hsa01100	Metabolic pathways		

## References

[1] Bartold P.M., Van Dyke T.E. (2017). Host modulation: Controlling the inflammation to control the infection. J. Periodontol..

[2] Kornman K.S. (2008). Mapping the pathogenesis of periodontitis: A new look. J. Periodontol..

[3] Berglundh T. (2019). Guidelines 4. Peri-Implant Health, Peri-Implant Mucositis, and Peri-Implantitis. European Federation of Periodontology, New Classification of Periodontal and Peri-Implant Diseases. https://www.efp.org/fileadmin/uploads/efp/Documents/Campaigns/New_Classification/Guidance_Notes/report-04.pdf.

[4] Baus-Domínguez M., Gómez-Díaz R., Corcuera-Flores J.R., Torres-Lagares D., Ruiz-Villandiego J.C., Machuca-Portillo G., Gutierrez-Pérez J.L., Serrera-Figallo M.A. (2019). Metallothioneins in failure of dental implants and periodontitis down syndrome patients. Genes.

[5] Baus-Domínguez M., Gómez-Díaz R., Torres-Lagares D., Corcuera-Flores J.R., Ruiz-Villandiego J.C., Machuca-Portillo G., Gutiérrez-Pérez J.L., Serrera-Figallo M.A. (2019). Differential expression of inflammation-related genes in down syndrome patients with or without periodontal disease. Mediat. Inflamm..

[6] Corcuera-Flores J.R., López-Giménez J., López-Jiménez J., López-Giménez A., Silvestre-Rangil J., Machuca-Portillo G. (2017). Four years survival and marginal bone loss of implants in patients with Down syndrome and cerebral palsy. Clin. Oral Investig..

[7] Silva C.R.S., Biselli-Périco J.M., Zampieri B.L., Silva W.A., De Souza J.E.S., Bürger M.C., Golini-Bertollo E.M., Pavarino E.C. (2016). Differential expression of inflammation-related genes in children with down syndrome. Mediat. Inflamm..

[8] Bassani D.G., Olinto M.T.A., Kreiger N. (2007). Periodontal disease and perinatal outcomes: A case-control study. J. Clin. Periodontol..

[9] Mao D., Epple H., Uthgenannt B., Novack D.V., Faccio R. (2006). PLCγ2 regulates osteoclastogenesis via its interaction with ITAM proteins and GAB2. J. Clin. Investig..

[10] Li S., Miller C.H., Giannopoulou E., Hu X., Ivashkiv L.B., Zhao B. (2014). RBP-J imposes a requirement for ITAM-mediated costimulation of osteoclastogenesis. J. Clin. Investig..

[11] Yu P., Constien R., Dear N., Katan M., Hanke P., Bunney T.D., Kunder S., Quintanilla-Martinez L., Huffstadt U., Schröder A. (2005). Autoimmunity and inflammation due to a gain-of-function mutation in phospholipase Cγ2 that specifically increases external Ca2+ entry. Immunity.

[12] Weber K.L., Doucet M., Shaner A., Hsu N., Huang D., Fogel J., Kominsky S.L. (2012). MIP-1δ activates NFATc1 and enhances osteoclastogenesis: Involvement of both PLCγ2 and NFκB signaling. PLoS ONE.

[13] Niu C., Xiao F., Yuan K., Hu X., Lin W., Ma R., Zhang X., Huang Z. (2017). Nardosinone suppresses RANKL-induced osteoclastogenesis and attenuates lipopolysaccharide-induced alveolar bone resorption. Front. Pharmacol..

[14] Lee J.-M., Park H., Noh A.L.S.M., Kang J.-H., Chen L., Zheng T., Lee J., Ji S., Jang C., Shin C.S. (2012). 5-Lipoxygenase mediates RANKL-induced osteoclast formation via the cysteinyl leukotriene receptor 1. J. Immunol..

[15] Kang J., Ting Z., Moon M., Sim J., Lee J., Doh K., Hong S., Cui M., Choi S., Chang H.W. (2015). 5-Lipoxygenase inhibitors suppress RANKL-induced osteoclast formation via NFATc1 expression. Bioorg. Med. Chem..

[16] Lopes D.E.M., Jabr C.L., Dejani N.N., Saraiva A.C., de Aquino S.G., Medeiros A.I., Junior C.R. (2017). Inhibition of 5-lipoxygenase (5-Lo) attenuates inflammation and bone resorption in lipopolysaccharide (Lps)-induced periodontal disease. J. Periodontol..

[17] Madeira M.F.M., Queiroz-Junior C.M., Corrêa J.D., Werneck S.M.C., Machado F.S., Cunha T.M., Garlet G.P., Teixeira M.M., Silva T.A., Souza D.G. (2017). The role of 5-lipoxygenase in Aggregatibacter actinomycetemcomitans induced alveolar bone loss. J. Clin. Periodontol..

[18] Hikiji H., Ishii S., Yokomizo T., Takato T., Shimizu T. (2009). A distinctive role of the leukotriene B 4 receptor BLT1 in osteoclastic activity during bone loss. Proc. Natl. Acad. Sci. USA.

[19] Dixit N., Wu D.J., Belgacem Y.H., Borodinsky L.N., Gershwin M.E., Adamopoulos I.E. (2014). Leukotriene B4 activates intracellular calcium and augments human osteoclastogenesis. Arthritis Res. Ther..

[20] Li X., Ding L., Wang Y., Li Z., Wang Q., Zhao Z., Zhao S., Wang H., Wu C., Mao N. (2020). Skeletal stem cell-mediated suppression on inflammatory osteoclastogenesis occurs via concerted action of cell adhesion molecules and osteoprotegerin. Stem Cells Transl. Med..

[21] Ren G., Zhao X., Zhang L., Zhang J., L’Huillier A., Ling W., Roberts A.I., Le A.D., Shi S., Shao C. (2010). Inflammatory cytokine-induced intercellular adhesion molecule-1 and vascular cell adhesion molecule-1 in mesenchymal stem cells are critical for immunosuppression. J. Immunol..

[22] Wu Y., Raymond B., Goossens P.L., Njamkepo E., Guiso N., Paya M., Touqui L. (2010). Type-IIA secreted phospholipase A2 is an endogenous antibiotic-like protein of the host. Biochimie.

[23] Weiss J.P. (2015). Molecular determinants of bacterial sensitivity and resistance to mammalian Group IIA phospholipase A2. Biochim. Biophys. Acta Biomembr..

[24] Dore E., Boilard E. (2019). Roles of secreted phospholipase A2 group IIA in inflammation and host defense. Biochim. Biophys. Acta Mol. Cell Biol. Lipids.

[25] Al-Attar A., Alimova Y., Kirakodu S., Kozal A., Novak M.J., Stromberg A.J., Orraca L., González-Martínez J., Martinez M., Ebersole J.L. (2018). Activation of Notch-1 in oral epithelial cells by P. gingivalis triggers the expression of the antimicrobial protein PLA2-IIA. Mucosal Immunol..

[26] Koduri R.S., Grönroos J.O., Laine V.J.O., Le Calvez C., Lambeau G., Nevalainen T.J., Gelb M.H. (2002). Bactericidal properties of human and murine groups I, II, V, X, and XII secreted phospholipases A 2. J. Biol. Chem..

